# The Global, Regional, and National Burden and Trends of Infective Endocarditis From 1990 to 2019: Results From the Global Burden of Disease Study 2019

**DOI:** 10.3389/fmed.2022.774224

**Published:** 2022-03-09

**Authors:** Huilong Chen, Yuan Zhan, Kaimin Zhang, Yiping Gao, Liyuan Chen, Juan Zhan, Zirui Chen, Zhilin Zeng

**Affiliations:** ^1^Department and Institute of Infectious Diseases, Tongji Medical College, Tongji Hospital, Huazhong University of Science and Technology, Wuhan, China; ^2^Department of Respiratory and Critical Care Medicine, National Clinical Research Center of Respiratory Disease, Tongji Medical College, Tongji Hospital, Huazhong University of Science and Technology, Wuhan, China; ^3^Department of Medical Engineering, Guangdong Provincial People's Hospital, Guangzhou, China; ^4^Department of Medical Ultrasound, Tongji Medical College, Tongji Hospital, Huazhong University of Science and Technology, Wuhan, China; ^5^Department of Obstetrics and Gynecology, Wuhan No.1 Hospital, Wuhan, China; ^6^Department of Dermatology, Tongji Medical College, Tongji Hospital, Huazhong University of Science and Technology, Wuhan, China; ^7^Second Clinical College, Tongji Medical College, Tongji Hospital, Huazhong University of Science and Technology, Wuhan, China

**Keywords:** infective endocarditis, 2019 GBD, incidence, mortality, disability-adjusted life years

## Abstract

**Introduction:**

Infective endocarditis (IE) presents with increasing incidence and mortality in some regions and countries, as well as serious socioeconomic burden. The current study aims to compare and interpret the IE burden and temporal trends globally and in different regions from 1990 to 2019.

**Methods:**

Data on the incidence, deaths and disability-adjusted life years (DALYs) caused by IE were extracted and analyzed from the Global Burden of Disease Study 2019. Estimated annual percentage changes (EAPC) were adopted to quantify the change trends of age-standardized rates (ASRs). Besides, potential contributors of serious IE burden were also evaluated including age, gender, social-demographic index (SDI), and age-standardized incident rate (ASIR) in 1990.

**Results:**

Globally, the number of IE cases and deaths has increased sharply during the past 30 years from 478,000 in 1990 to 1,090,530 in 2019 and from 28,750 in 1990 to 66,320 in 2019, and both presented an upward temporal trend annually (EAPC:1.2 for incidence and 0.71 for death). However, the EAPC of age-standardized DALYs demonstrated a negative temporal trend despite increasing DALYs from 1,118,120 in 1990 to 1,723,590 in 2019. Moreover, older patients and men were more severely affected. Meanwhile, different SDI regions had different disease burdens, and correlation analyses indicated that SDI presented a positive association with ASIR (R = 0.58, *P* < 0.0001), no association with age-standardized death rate (R = −0.06, *P* = 0.10), and a negative association with age-standardized DALYs (R = −0.40, *P* < 0.0001). In addition, the incidence of IE increased in most countries during the past 30 years (190 out of 204 countries). However, the change trends of deaths and DALYs were heterogeneous across regions and countries. Finally, we discovered positive associations of the EAPC of ASRs with the SDI in 2019 among 204 countries and territories but few associations with the ASIR in 1990.

**Conclusion:**

Generally, the global burden of IE is increasing, and there is substantial heterogeneity in different genders, ages and regions, which may help policy-makers and medical staff respond to IE and formulate cost-effective interventional measures.

## Introduction

Infective endocarditis (IE) is a life-threatening cardiac infection and is predisposed to occur in some individuals with multiple cardiac valve conditions (1), with an annual incidence of ~3–10 per 100,000 people (2). Globally, IE remains a highly lethal disease, with the overall mortality remaining at ~25% (2, 3). Specifically, a population-based survey in France suggested that the age-standardized and sex-standardized annual incidence of IE was 33.8 cases per million inhabitants and the overall in-hospital mortality rate was 22.7% (4). A similarly high mortality of IE was likewise reported in some developing countries (5). Furthermore, the burden of IE was heavy and the inflation-adjusted expenditure on IE hospitalizations in the United States rose significantly from $1.56 billion in 2003 to $2.34 billion in 2016 (6). Therefore, increased efforts to reduce the burden of IE seem logical and needed (7).

The diagnosis and treatment of IE have developed rapidly but differed greatly in different regions and countries, resulting in the same substantial differences in disease burden worldwide. The contributing risk factors for IE morbidity were diverse, including rheumatic heart disease, prosthetic valve or cardiac device use, intravenous drug use and other blood-borne microbial infections (8, 9). Meanwhile, the complex and fickle clinical presentations and courses of IE brought about tremendous diagnostic and therapeutic challenges, and there were various response capacities among different regions (10). Hidden cases may remain undetected in regions that have not kept up with updated diagnostic technology such as ^18^F-FDG PET scanning for those with implanted device and valves (8, 11) with higher diagnostic sensitivity (12). Furthermore, therapy under a multispecialty team including an infectious disease specialist, cardiologist, and cardiac surgeon has been reported to reduce the time to surgery and both operative and long-term mortality (2, 13). All these elements result in the heterogeneity of the IE burden between relatively high and low income regions.

A systematic review of the 2010 GBD study provided a plain epidemiological estimate of IE in some areas and countries (14) and demonstrated the significant morbidity and mortality of IE. However, as of now, there lacks a comprehensive and thorough report presenting the disease burden of IE at the global level. Here, by analyzing the data from the 2019 GBD study, we aim to summarize the incidence, death rate, disability-adjusted life years (DALYs) and the corresponding trend changes for IE in 204 countries and territories, 21 geographic regions and 5 SDI regions, and determine the association between IE burden and SDI.

## Methods

### Data Acquisition

Global Burden of Disease 2019 (GBD 2019) provided the information about the burdens of 369 diseases and injuries along with 87 risk factors in the globe, different geographic areas, and 204 countries and territories (15, 16) and the detailed introduction was shown in Supplementary File 1, which aimed at quantifying the relative magnitude of health loss due to risk factors, injuries and diseases by age, gender, SDI (sociodemographic index) and geographic location at a specific point in time. Data on infective endocarditis burden from 1990 to 2019 including its incidence, death, DALYs, and their corresponding age-standardized rates (ASRs), were acquired from the Global Health Data Exchange GBD Results Tool (http://ghdx.healthdata.org/gbd-results-tool) (date of data extraction, 21 April of 2021). Here, as the case definition of 2019 GBD stated, included cases of IE were mainly based on clinical diagnosis (15). Meanwhile, the information about the distributions of gender and age was also obtained. The rates were standardized according to the GBD world population and were reported per 100,000 person-years. The GBD 2019 used various interrelated parameters to measure population health loss, including number of cases and incidence, number of deaths and mortality, and DALYs. As for the current report, we used the GBD Results Tool to extract estimates and their 95% certainty intervals (CIs) for incidence of cases, deaths, and DALYs as measures of IE burden from 1990 to 2019 by region and country. To better exhibit the age distribution of IE burden, IE patients were classified into 5 groups, namely those aged under 5 years, from 5 to 14 years, from 15 to 49 years and from 50 to 69 years, as well as above 70 years. Social developmental index (SDI) was a composite indicator of total fertility, per capita income, and the years of schooling, which was formulated to reflect the social developmental degree (17). The value ranged from 0 to 1, where 0 represented the fewest years of schooling, lowest per capita income, and highest fertility, whereas 1 represented the opposite. Previous studies have reported that the incidence and mortality of diseases might be affected by the social developmental degree. Base on that, the 204 countries and territories were classified into 5 groups according to the SDI quintile (low-SDI, low-middle-SDI, middle-SDI, high-middle-SDI, and high-SDI) to explore the association between IE burden and social development degrees in different regions.

### Statistical Analysis

The number of cases and deaths as well as age-standardized incidence, age-adjusted mortality, and age-standardized DALYs were the main metrics characterizing the IE burden and compared at the global, regional, and country levels. CIs were calculated from 1,000 draw-level estimates for each parameter and 95% CIs were defined by the 25th and 975th values of the ordered 1000 estimates; a 95% CI excluding 0 was considered statistically significant. ASR were calculated on the basis of the following formula: $ASR=\frac{\overset{A}{\underset{i=1}{\sum }}a_{i}w_{i}}{\overset{A}{\underset{i=1}{\sum }}w_{i}}\times$100,000. The ASR (per 100,000 population) was equal to the sum of the product of the specific age ratio (a_i_) in age group i and the number (or weight) (w_i_) of the selected reference standard population group i divided by the sum of number (or weight) of the standard population. To investigate the dynamic changes of IE burden, we further calculated the estimated annual percentage change (EAPC) to delineate the temporal trend in different ASRs of IE burden (18). We performed a regression model fitting the natural logarithm of the ASR with the calendar year, namely, ln (ASR) = α + β^*^ calendar year + ε, to estimate the EAPC with its 95% CI based on the formula of 100 × (exp (β) – 1). If the EAPC value and its 95% CI were both above zero, the change trend of ASR was considered upwards, and vice versa. Otherwise, the ASR was considered stable over time (19–21). In addition, we examined the shape of the association between ASRs of infective endocarditis and the SDI using the fit spline models (22). Finally, we used Pearson's correlation test to analyze the correlation between EAPC and SDI in 2019 or the age-standardized incident rate (ASIR) in 1990 in 204 countries and territories. The maps were made using ECharts software. All statistical analyses were performed using GraphPad Prism 8 software and SPSS statistics 22.

## Results

### Incidence of Infective Endocarditis

**Figure 1:**
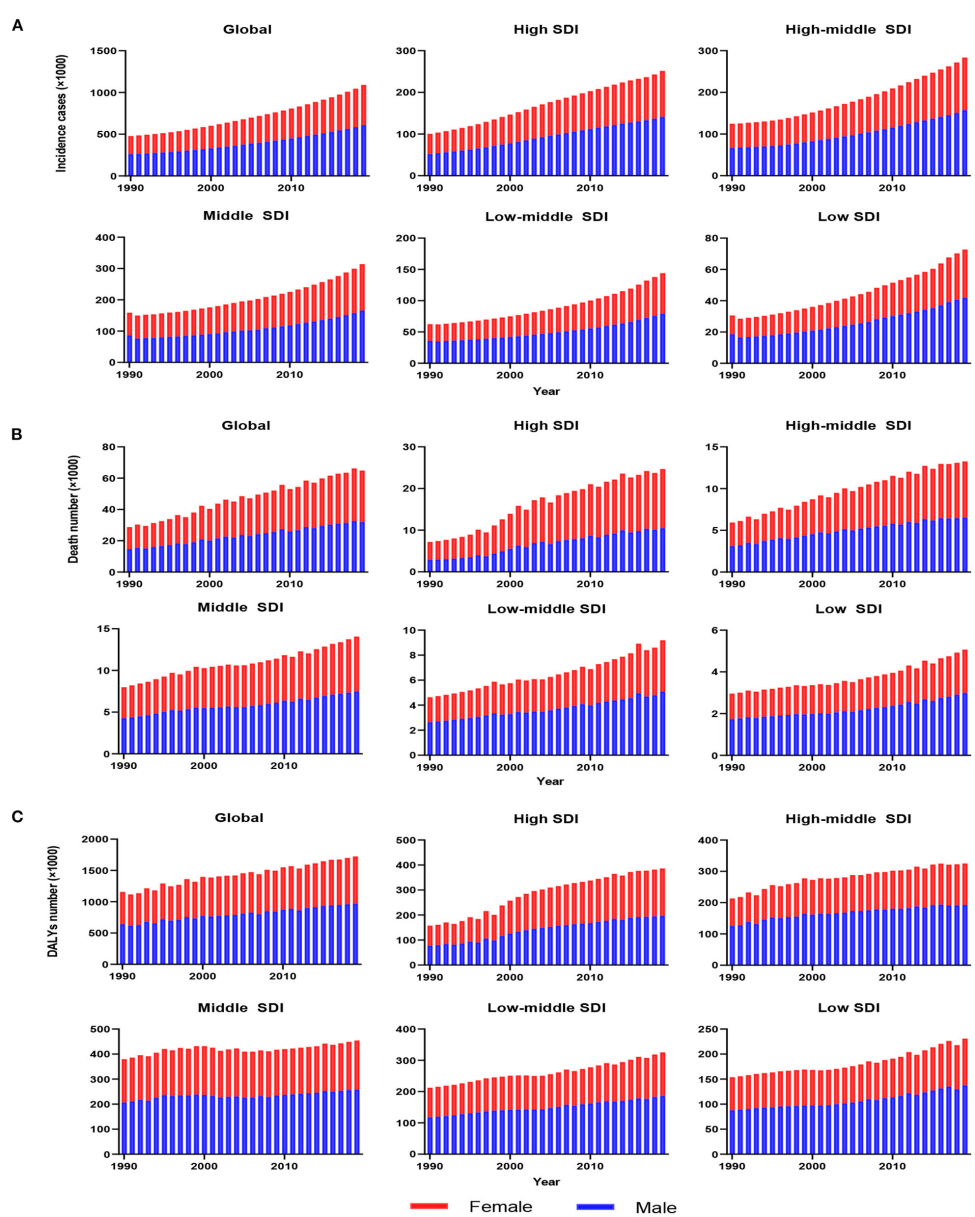
The change trends of infective endocarditis incidences, deaths and DALYs from 1990 to 2019. The change trends of incidences **(A)**; the change trends of deaths **(B)**; and the change trends of DALYs **(C)**. Blue bars represent males, and red bars represent females. DALYs, disability-adjusted life-years; SDI, social-demographic index.

**Figure 2:**
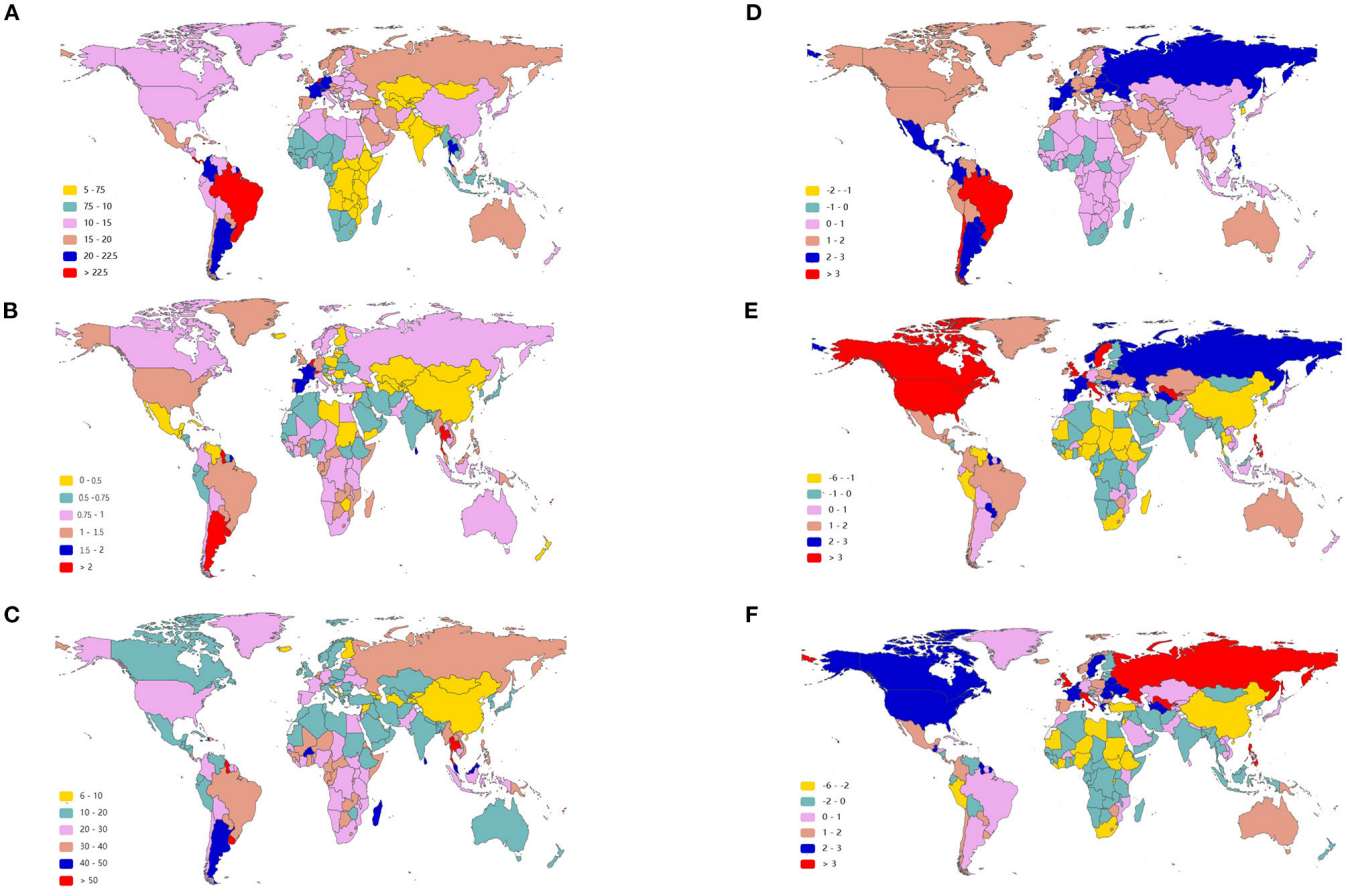
The age-standardized rate of infective endocarditis in 2019 and EAPC of IE ASRs from 1990 to 2019 in 204 countries and territories. The ASIR of IE around the world in 2019 **(A)**; The ASDR of IE across the world in 2019 **(B)**; The age-standardized DALY rate of IE throughout the world in 2019 **(C)**; The EAPC of ASIR **(D)**, ASDR **(E)**, and age-standardized DALY rate **(F)** in the past 30 years. IE, infective endocarditis; ASIR, age-standardized incident rate; ASDR, age-standardized death rate; DALYs, disability-adjusted life-years; EAPC, estimated annual percentage change; ASRs, age-standardized rates.

**Figure 3:**
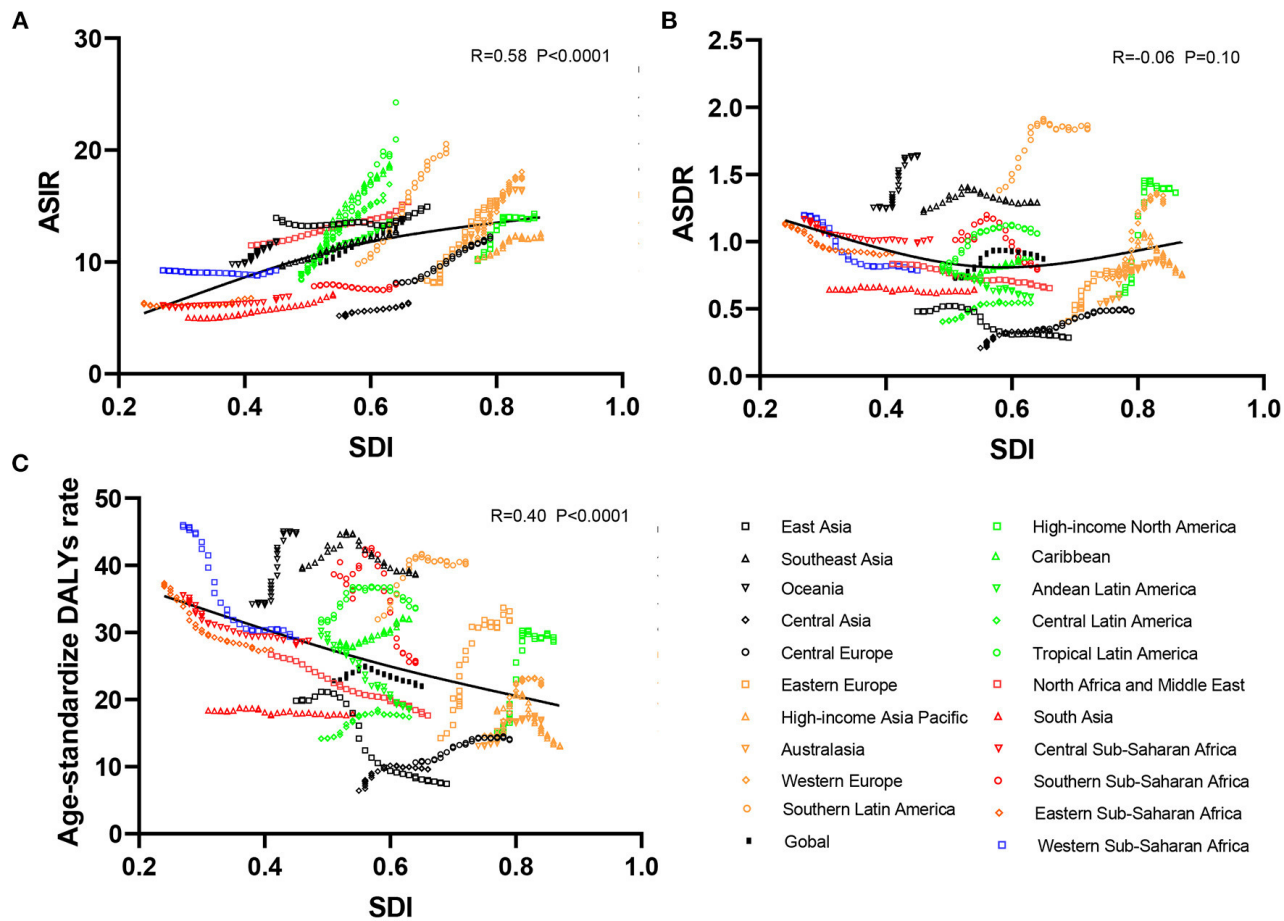
Correlation analyses between ASRs of infective endocarditis and SDI in 21 regions from 1990 to 2019. The SDI was positively correlated with the ASIR **(A)** and negatively correlated with the age-standardized DALY rate **(C)** but not correlated with ASDR **(B)** in 21 regions from 1990 to 2019. ASRs, age-standardized rates; ASIR, age-standardized incident rate; ASDR, age-standardized death rate; DALYs, disability-adjusted life-years; SDI, social-demographic index.

As presented in Table 1, the incidence of infective endocarditis reached 1,090,530 in 2019 from 478,000 in 1990. The age-standardized incident rate (ASIR) increased from 9.91 per 100,000 population in 1990 to 13.80 in 2019, which showed an upward trend worldwide (EAPC, 1.2; 95% CI: 1.16–1.24). Globally, the incidences in different areas were divergent. In the past 30 years, the incidence of IE in different SDI (sociodemographic index) regions rose gradually, and the incidence in males was higher than that in females (males: EAPC, 1.4; 95% CI: 1.36–1.45; females: EAPC, 0.96; 95% CI, 0.93–1.00) (Figure 1A, Table 1). In 1990, the high-middle SDI region had the most ASIR (11.34 per 100,000 population [95% CI: 9.44–13.59]), the middle SDI region had the most incidence cases (159,200 [95% CI: 130,200–191,000]), while the incidence case or ASIR of the low SDI region was the lowest (30,590 [95% CI: 24,430–38,070]; 6.29 [95% CI: 5.23–7.50]) (Table 1). In 2019, the incidence of IE almost doubled, and the ASIR increased by 1–4 per 100,000 population compared to 1990, although the distribution of incidence and ASIR remained unchanged (Table 1). Among the 21 regions divided according to geographical characteristics, East Asia had the most ASIR in 1990 and was replaced by Tropical Latin America in 2019 (Figure 2A, Table 1). The EAPC of the ASIR in our datasets was positive in most regions apart from Western Sub-Saharan Africa and Southern Sub-Saharan Africa, which signified that the IE incidence was on the rise. South America and Western Europe had high growth rates, while the growth rates in South Asia, Central Asia and Sub-Saharan Africa were relatively low (Figure 2D). At the country level, the top three countries with the highest incidence of IE were Saint Lucia (35.83 [95% CI: 31.22–40.41]), Grenada (32.26 [95%CI: 28.22–36.50]), and Barbados (31.11 [95%CI: 27.21–35.39]) (Figure 2A, Supplementary Data Sheet 3). In addition, the fastest growth of the ASIR was in Brazil (EAPC: 3.26), Chile (EAPC: 3.25), and Russian Federation Russian (EAPC: 2.98) (Figure 2D, Supplementary Data Sheet 3). In Figure 3A, the ASIR and SDI are positively correlated (R = 0.58, *P* < 0.0001), which seems to verify our results above. In other words, the ASIR seems to be higher in relatively developed regions.

**Table 1:** The incidence of infective endocarditis in 1990/2019 and temporal trends.

**Characteristics**	**1990**		**2019**		**1990–2019**
	**Incident cases No × 10^**3**^ (95% CI)**	**ASIR/10^**5**^ No. (95% CI)**	**Incident cases No × 10^**3**^ (95% CI)**	**ASIR/10^**5**^ No. (95% CI)**	**EAPC No. (95% CI)**
Overall	478.00 (393.39~572.42)	9.91 (8.24~11.84)	1,090.53 (913.50~1,296.29)	13.80 (11.59~16.34)	1.2 (1.16~1.24)
Sex					
Male	260.80 (215.67~310.47)	11.04 (9.28~13.11)	610.10 (514.07~719.32)	16.20 (13.75~18.918)	1.4 (1.36~1.45)
Female	217.20 (178.42~262.26)	8.84 (7.31~10.63)	480.43 (401.56~575.14)	11.62 (9.67~13.83)	0.96 (0.93~1)
Socio-demographic factor					
High SDI	100.54 (83.61~121.96)	11.01 (9.14~13.37)	251.57 (215.00~296.06)	15.85 (13.56~18.53)	1.25 (1.09~1.41)
High-middle SDI	124.76 (102.74~150.52)	11.34 (9.44~13.59)	283.71 (235.85~341.81)	15.86 (13.24~18.93)	1.26 (1.21~1.31)
Middle SDI	159.20 (130.20~191.00)	10.50 (8.75~12.50)	314.02 (260.10~379.41)	13.22 (11.01~15.77)	0.99 (0.89~1.09)
Low-middle SDI	62.64 (50.33~76.48)	6.47 (5.31~7.78)	144.05 (117.96~174.20)	9.33 (7.79~11.16)	1.31 (1.19~1.43)
Low SDI	30.59 (24.43~38.07)	6.29 (5.23~7.50)	72.75 (58.02~89.54)	7.39 (6.13~8.809)	0.81 (0.65~0.97)
Region					
East Asia	157.04 (128.24~188.45)	13.93 (11.51~16.57)	268.46 (218.13~336.02)	14.94 (12.35~18.11)	0.25 (0.13~0.36)
Southeast Asia	33.53 (27.93~39.87)	9.60 (8.20~11.17)	80.20 (67.56~93.77)	12.84 (10.96~14.86)	0.96 (0.91~1.01)
Oceania	0.47 (0.38~0.56)	9.80 (8.32~11.39)	1.17 (0.99~1.36)	11.86 (10.27~13.62)	0.72 (0.68~0.75)
Central Asia	3.12 (2.43~3.91)	5.19 (4.11~6.47)	5.42 (4.26~6.79)	6.35 (5.12~7.83)	0.76 (0.71~0.81)
Central Europe	10.81 (8.78~13.36)	8.15 (6.60~10.05)	21.03 (17.44~25.38)	12.28 (10.21~14.68)	1.63 (1.52~1.74)
Eastern Europe	21.13 (17.16~26.03)	8.49 (6.91~10.37)	41.87 (34.67~50.42)	15.456 (12.88~18.32)	2.71 (2.51~2.92)
High-income Asia Pacific	18.73 (15.26~23.10)	10.22 (8.39~12.44)	43.46 (36.12~52.58)	12.54 (10.35~15.15)	0.6 (0.45~0.74)
Australasia	2.50 (2.06~3.01)	11.24 (9.32~13.52)	7.17 (5.98~8.49)	16.46 (13.78~19.51)	1.38 (1.24~1.51)
Western Europe	53.68 (44.46~65.37)	11.29 (9.30~13.75)	136.27 (115.90~159.92)	18.06 (15.32~21.34)	1.72 (1.6~1.84)
Southern Latin America	4.52 (3.83~5.29)	9.83 (8.41~11.43)	16.03 (13.78~18.55)	20.55 (17.67~23.68)	2.62 (2.5~2.75)
High-income North America	31.03 (25.65~37.69)	10.11 (8.32~12.27)	77.03 (66.36~89.25)	14.31 (12.38~16.50)	1.11 (0.85~1.38)
Caribbean	3.48 (2.93~4.10)	11.14 (9.42~13.09)	9.21 (7.88~10.69)	18.72 (16.06~21.74)	1.8 (1.65~1.95)
Andean Latin America	2.51 (2.11~2.96)	8.79 (7.43~10.39)	8.01 (6.65~9.53)	13.58 (11.31~16.12)	1.46 (1.37~1.55)
Central Latin America	10.79 (8.63~13.30)	8.48 (6.97~10.39)	40.49 (33.49~49.46)	16.93 (14.03~20.44)	2.22 (2.03~2.41)
Tropical Latin America	10.37 (8.45~12.52)	8.42 (7.04~10.04)	57.44 (46.78~70.21)	24.25 (19.88~29.56)	3.25 (3.13~3.38)
North Africa and Middle East	33.37 (27.16~40.60)	11.47 (9.62~13.59)	81.53 (67.01~97.90)	15.35 (12.89~18.20)	0.96 (0.92~1)
South Asia	44.70 (34.84~56.22)	5.02 (4.07~6.12)	115.83 (92.25~142.61)	7.11 (5.79~8.64)	1.25 (1.12~1.38)
Central Sub-Saharan Africa	2.85 (2.28~3.60)	6.135 (5.15~7.26)	7.36 (5.78~9.16)	6.89 (5.75~8.20)	0.4 (0.29~0.51)
Eastern Sub-Saharan Africa	9.80 (7.90~12.20)	6.33 (5.32~7.48)	22.59 (17.68~28.26)	6.77 (5.59~8.06)	0.18 (0.06~0.3)
Southern Sub-Saharan Africa	3.49 (2.80~4.24)	7.83 (6.52~9.28)	5.69 (4.59~6.91)	8.09 (6.65~9.70)	−0.12 (−0.22~-0.02)
Western Sub-Saharan Africa	20.07 (15.87~25.04)	9.25 (7.71~10.95)	44.28 (35.15~55.81)	9.24 (7.68~11.04)	−0.11 (−0.16~-0.05)

*ASIR, age-standardized incident rate per 100,000 population; SDI, social-demographic index; EAPC, estimated annual percentage change*.

### Death of Infective Endocarditis

Worldwide, there were 28,750 deaths in 1990 and 66,320 deaths in 2019 (Table 2). The age-standardized death rate (ASDR) (Figure 2B) and their changing trends varied among different countries (Figure 2E). Although the number of deaths in different SDI areas was increasing, their changing trend, namely, EAPC of ASDR, was not always rising. Moreover, females had more deaths from IE than males in 2019 (males: death, 32,630; 95% CI, 22,300–37,640; females: death, 33,690; 95% CI, 21,430–39,180) (Figure 1B, Table 2).

**Table 2:** The deaths due to infective endocarditis in 1990/2019 and temporal trends.

**Characteristics**	**1990**		**2019**		**1990–2019**
	**Death No × 10^**3**^ (95% CI)**	**ASDR/10^**5**^ No. (95% CI)**	**Death No × 10^**3**^ (95% CI)**	**ASDR/10^**5**^ No. (95% CI)**	**EAPC No. (95% CI)**
Overall	28.75 (24.37~35.7)	0.73 (0.63~0.93)	66.32 (46.21~75.86)	0.87 (0.59~1)	0.71 (0.44~0.98)
Sex					
Male	14.72 (12.06~18.7)	0.77 (0.65~1.02)	32.63 (22.3~37.64)	0.94 (0.62~1.09)	0.79 (0.57~1.01)
Female	14.03 (10.94~18.81)	0.66 (0.53~0.91)	33.69 (21.43~39.18)	0.78 (0.5~0.91)	0.66 (0.35~0.96)
Socio-demographic factor					
High SDI	7.19 (5.9~11.49)	0.71 (0.58~1.12)	24.68 (11.73~30.81)	1.16 (0.58~1.43)	2.01 (1.51~2.51)
High-middle SDI	5.94 (4.99~7.71)	0.6 (0.5~0.78)	13.26 (9.19~15.08)	0.71 (0.49~0.8)	0.62 (0.38~0.86)
Middle SDI	8 (6.54~9.53)	0.69 (0.56~0.84)	14.07 (12.02~17.64)	0.62 (0.52~0.77)	−0.62 (−0.76~−0.48)
Low-middle SDI	4.64 (3.5~5.48)	0.66 (0.52~0.78)	9.19 (7.49~11.1)	0.67 (0.55~0.82)	−0.06 (−0.13~0.01)
Low SDI	2.96 (1.83~4.07)	0.9 (0.55~1.38)	5.07 (3.72~6.61)	0.79 (0.55~1.06)	−0.56 (−0.64~−0.48)
Region					
East Asia	4.36 (2.73~5.34)	0.48 (0.31~0.58)	4.91 (3.75~5.83)	0.29 (0.21~0.34)	−2.4 (−2.78~−2.02)
Southeast Asia	3.54 (2.83~4.91)	1.22 (0.97~1.69)	7.48 (6.03~10.35)	1.29 (1.04~1.76)	0 (−0.16~0.16)
Oceania	0.04 (0.02~0.06)	1.25 (0.78~2.25)	0.12 (0.08~0.16)	1.64 (1.13~2.26)	1.24 (1.11~1.37)
Central Asia	0.1 (0.08~0.13)	0.21 (0.16~0.27)	0.24 (0.2~0.32)	0.33 (0.28~0.44)	1.69 (1.44~1.94)
Central Europe	0.47 (0.41~0.69)	0.34 (0.3~0.5)	0.93 (0.56~1.14)	0.48 (0.28~0.59)	1.39 (1.15~1.63)
Eastern Europe	1.01 (0.77~1.83)	0.39 (0.3~0.7)	2.19 (1.59~2.64)	0.78 (0.56~0.95)	2.44 (2.05~2.83)
High-income Asia Pacific	1.37 (0.93~1.6)	0.79 (0.53~0.92)	4.42 (1.82~5.96)	0.75 (0.36~0.97)	−0.69 (−1.09~−0.28)
Australasia	0.12 (0.1~0.18)	0.54 (0.46~0.79)	0.43 (0.2~0.56)	0.82 (0.39~1.04)	1.71 (1.38~2.04)
Western Europe	4.12 (3.15~7.55)	0.72 (0.56~1.3)	13.79 (6.58~17.12)	1.28 (0.63~1.57)	2.59 (2.15~3.03)
Southern Latin America	0.59 (0.41~0.75)	1.38 (0.94~1.77)	1.53 (1.18~1.8)	1.84 (1.42~2.16)	0.78 (0.48~1.07)
High-income North America	2.14 (1.63~3.84)	0.61 (0.46~1.08)	8.98 (4.33~11.04)	1.36 (0.68~1.65)	3.26 (2.43~4.09)
Caribbean	0.22 (0.18~0.29)	0.75 (0.65~0.97)	0.44 (0.35~0.54)	0.88 (0.71~1.08)	0.75 (0.69~0.81)
Andean Latin America	0.22 (0.16~0.28)	0.8 (0.59~0.94)	0.33 (0.25~0.43)	0.59 (0.44~0.76)	−1.11 (−1.18~−1.03)
Central Latin America	0.43 (0.38~0.61)	0.4 (0.35~0.58)	1.29 (0.89~1.58)	0.54 (0.38~0.67)	1.15 (0.92~1.38)
Tropical Latin America	0.94 (0.8~1.47)	0.82 (0.7~1.35)	2.51 (1.77~2.82)	1.06 (0.74~1.19)	1.04 (0.78~1.3)
North Africa and Middle East	1.73 (1.25~2.12)	0.83 (0.64~0.99)	2.74 (2.07~3.29)	0.65 (0.51~0.79)	−0.85 (−0.92~−0.78)
South Asia	3.79 (2.95~4.77)	0.64 (0.51~0.79)	8.77 (6.93~10.89)	0.64 (0.51~0.8)	−0.12 (−0.2~−0.04)
Central Sub-Saharan Africa	0.32 (0.18~0.51)	1.17 (0.58~2.03)	0.61 (0.35~0.97)	1.02 (0.56~1.68)	−0.58 (−0.65~−0.51)
Eastern Sub-Saharan Africa	1.13 (0.61~1.81)	1.14 (0.55~2.05)	1.83 (1.07~2.89)	0.91 (0.5~1.49)	−0.9 (−1~−0.8)
Southern Sub-Saharan Africa	0.37 (0.25~0.44)	1.01 (0.7~1.2)	0.48 (0.39~0.61)	0.79 (0.65~1)	−1.11 (−1.5~−0.71)
Western Sub-Saharan Africa	1.74 (0.91~2.45)	1.2 (0.73~1.76)	2.29 (1.76~3.05)	0.78 (0.61~1.02)	−1.77 (−1.99~−1.55)

*ASDR, age-standardized death rate per 100,000 population; SDI, social-demographic index; EAPC, estimated annual percentage change*.

Although the low SDI region had the lowest number of deaths (2,960 [95% CI: 1,830–4,070]) in 1990, the mortality rate (ASDR, 0.9 per 100,000 population [95% CI: 0.55–1.38]) was the highest, and it continued to be the region with the lowest number of deaths (5,070 [95% CI: 3,720–6,610]) in 2019. The high SDI region had the highest number of deaths (24,680 [95% CI: 11,730–30,810]) and the ASDR (1.16 [95% CI: 0.58–1.43]) in 2019 (Table 2). The EAPC of ASDR from 1990 to 2019 showed significant differences in ASDR between different SDI regions in the past 30 years. The ASDR of high SDI region grew fastest (EAPC, 2.01 [95%CI: 1.51–2.51]) while the ASDR of middle (EAPC, −0.62 [95%CI: −0.76–−0.48]) and low SDI regions (EAPC, −0.56 [95%CI: −0.64–−0.48]) was decreasing (Table 2), which was different from the continuous increase of death number in each SDI region in the past 30 years as shown in Figure 1B. Between the 21 regions, Oceania had the lowest number of deaths in both 1990 and 2019, while the region with the highest deaths changed from East Asia in 1990 to Western Europe in 2019 (Table 2). Furthermore, southern Latin America and Oceania had the highest and second highest ASDRs in 1990 and 2019, respectively (Figure 2B, Table 2). Fascinatingly, East Asia, which had the most deaths in 1990, held the lowest ASDR in 2019 and declined fastest in ASDR from 1990 to 2019 (EAPC, −2.4 [95% CI: −2.78–−2.02]). The EAPC of ASDR of different regions was quite heterogeneous. High-income North America, Western Europe and Eastern Europe had relatively high growth rates, while East Asia and Western Sub-Saharan Africa had relatively high decline rates (Figure 2E, Table 2). Furthermore, among different countries, three island nations stood the highest ASDR, namely Kiribati (5.46 [95% CI: 3.51–8.32]), Fiji (3.27 [95% CI: 2.14–4.35]) and American Samoa (3.13 [95% CI: 1.74–4.32]), which suggested us that improving medical technology and conditions in the relatively undeveloped regions might alleviate death rate and disease burden of IE (Figure 2B, Supplementary Data Sheet 3). However, the top three of upward trend in ASDRs were Taiwan, Italy, and Switzerland with EAPCs of 8.10, 5.79, and 5.24 respectively (Figure 2E, Supplementary Data Sheet 3). The ASDR was not correlated with the SDI (R = −0.06, *P* = 0.10), which means there was no specific relationship between the ASDR and SDI even if the high SDI region had the most deaths and ASDR in 2019 (Figure 3B, Table 2).

### DALYs of Infective Endocarditis

The DALYs attributable to IE were on the rise globally from 1,118,120 in 1990 to 1,723,590 in 2019, with a preponderance for males (Table 3, Figure 1C). However, the age-standardized DALYs changed from 22.78 per 100,000 population in 1990 to 21.93 in 2019 around the world, and the EAPC of age-standardized DALYs was negative in both sexes. The middle SDI region obtained the highest DALYs in both 1990 and 2019, as the low SDI region had the lowest DALYs. Moreover, the low SDI region retained the highest age-standardized DALYs in 1990 and 2019. Regionally, the DALYs were found to be highest in East Asia in 1990 (225,160 [95% CI: 123,240–291,150]) and South Asia in 2019 (287,100 [95% CI: 225,890–360,890]). The age-standardized DALYs of Southern Latin America, Oceania and Southeast Asia were relatively high in both 1990 and 2019, whereas those of East Asia, Central Asia and Central Europe were relatively low (Table 3, Figure 2C). Interestingly, Eastern Europe (2.81 [95% CI: 2.35–3.27]) and High-income North America (2.66 [95% CI: 2.01–3.32]) held the highest EAPC of age-standardized DALYs (Table 3, Figure 2F).

**Table 3:** The DALYs of infective endocarditis in 1990/2019 and temporal trends.

**Characteristics**	**1990**		**2019**		**1990–2019**
	**DALYs No × 10^**3**^ (95% CI)**	**Age standardized DALYs/10^**5**^ No. (95% CI)**	**DALYs No × 10^**3**^ (95% CI)**	**Age standardized DALYs/10^**5**^ No. (95% CI)**	**EAPC No. (95% CI)**
Overall	1,118.12 (836.79~1,318.66)	22.78 (17.98~26.97)	1,723.59 (1,355.67~1,935.25)	21.93 (17.17~24.6)	−0.21 (−0.35~-0.08)
Sex					
Male	617.35 (463.03~749.04)	25.41 (20.09~31.37)	972.61 (735.86~1,123.53)	25.46 (19.06~29.12)	−0.05 (−0.18~0.07)
Female	500.77 (319.04~627.69)	20 (13.53~25.53)	750.98 (536.88~870.75)	18.33 (13.23~21.33)	−0.41 (−0.56~−0.25)
Socio-demographic factor					
High SDI	157.8 (131.72~234.56)	16.27 (13.51~24)	386.32 (212.21~461.4)	22.33 (12.61~26.23)	1.32 (0.95~1.7)
High-middle SDI	213.6 (170.09~253.17)	19.46 (15.45~23.03)	325.01 (238.95~365.72)	18.38 (13.65~20.7)	−0.42 (−0.62~−0.23)
Middle SDI	379.69 (280.63~449.48)	24.34 (19.13~28.65)	454.53 (390.82~582.66)	18.55 (15.94~23.45)	−1.2 (−1.34~−1.07)
Low-middle SDI	212.42 (138.86~271.28)	21.77 (16.16~25.98)	325.38 (262.88~395.76)	20.48 (16.51~24.91)	−0.32 (−0.39~−0.25)
Low SDI	153.84 (79.37~223.34)	31.18 (19.43~43.54)	230.95 (173.53~299.55)	25.3 (18.62~32.83)	−0.82 (−0.92~−0.72)
Region					
East Asia	225.16 (123.24~291.15)	0.48 (0.31~0.58)	127.6 (100.95~153.97)	0.29 (0.21~0.34)	−4.4 (−4.91~−3.88)
Southeast Asia	151.03 (122.05~217.56)	1.22 (0.97~1.69)	255.83 (208.03~359.59)	1.29 (1.04~1.76)	−0.26 (−0.46~−0.06)
Oceania	1.55 (1.03~2.38)	1.25 (0.78~2.25)	4.61 (3.1~6.41)	1.64 (1.13~2.26)	1.26 (1.13~1.4)
Central Asia	3.77 (3.06~4.52)	0.21 (0.16~0.27)	8.27 (6.95~11.3)	0.33 (0.28~0.44)	1.35 (1.05~1.65)
Central Europe	14.36 (12.75~20.56)	0.34 (0.3~0.5)	23.26 (13.6~29.04)	0.48 (0.28~0.59)	1.16 (0.94~1.38)
Eastern Europe	35.68 (27.36~65.37)	0.39 (0.3~0.7)	79.48 (53.86~97.14)	0.78 (0.56~0.95)	2.81 (2.35~3.27)
High-income Asia Pacific	33.18 (22.99~38.31)	0.79 (0.53~0.92)	56.07 (30.42~69.53)	0.75 (0.36~0.97)	−1.72 (−1.99~−1.44)
Australasia	2.93 (2.53~4.18)	0.54 (0.46~0.79)	7.44 (3.85~9.11)	0.82 (0.39~1.04)	1.05 (0.8~1.3)
Western Europe	77.71 (61.59~135.36)	0.72 (0.56~1.3)	193.13 (100.18~232.92)	1.28 (0.63~1.57)	1.91 (1.51~2.32)
Southern Latin America	14.77 (11.08~19.2)	1.38 (0.94~1.77)	31.78 (25.79~39.14)	1.84 (1.42~2.16)	0.58 (0.34~0.83)
High-income North America	49.8 (40.43~82.72)	0.61 (0.46~1.08)	159.45 (85.9~187.88)	1.36 (0.68~1.65)	2.66 (2.01~3.32)
Caribbean	9.71 (7.34~14.09)	0.75 (0.65~0.97)	15.38 (11.98~19.67)	0.88 (0.71~1.08)	0.54 (0.47~0.61)
Andean Latin America	11.56 (7.57~15.8)	0.8 (0.59~0.94)	11.27 (8.61~14.62)	0.59 (0.44~0.76)	−1.8 (−1.88~−1.72)
Central Latin America	20.35 (17.53~27.1)	0.4 (0.35~0.58)	42.52 (29.45~52.97)	0.54 (0.38~0.67)	0.9 (0.68~1.12)
Tropical Latin America	43.84 (35.45~65.78)	0.82 (0.7~1.35)	79.11 (61.28~94.86)	1.06 (0.74~1.19)	0.29 (0.08~0.5)
North Africa and Middle East	82.57 (45.09~114.67)	0.83 (0.64~0.99)	91.25 (65.94~111.62)	0.65 (0.51~0.79)	−1.43 (−1.49~−1.36)
South Asia	150.64 (111.05~200.69)	0.64 (0.51~0.79)	287.1 (225.89~360.89)	0.64 (0.51~0.8)	−0.15 (−0.21~−0.09)
Central Sub-Saharan Africa	14.47 (7.6~22.28)	1.17 (0.58~2.03)	23.75 (14.08~36.48)	1.02 (0.56~1.68)	−0.83 (−0.89~−0.77)
Eastern Sub-Saharan Africa	54.04 (28.51~79.44)	1.14 (0.55~2.05)	77.69 (48.46~117.8)	0.91 (0.5~1.49)	−1.2 (−1.3~−1.1)
Southern Sub-Saharan Africa	17.38 (11.47~20.9)	1.01 (0.7~1.2)	18.77 (15.33~23.82)	0.79 (0.65~1)	−1.69 (−2.15~−1.23)
Western Sub-Saharan Africa	103.62 (44.84~152.58)	1.2 (0.73~1.76)	129.82 (95.04~177.18)	0.78 (0.61~1.02)	−1.89 (−2.12~−1.66)

*DALY, disability-adjusted life-years; SDI, social-demographic index; EAPC, estimated annual percentage change*.

As shown in Figures 2B,C and Supplementary Data Sheet 3, the distribution of countries with age-standardized DALYs and ASDR in 2019 was similar. At the various country levels, the top 3 countries in the ranking of age-standardized DALYs remained Kiribati (166,694 [95% CI: 115,410–244,271]), Fiji (90,976 [95% CI: 59,134–123,393]) and American Samoa (81,443 [95% CI: 45,470–114,493]). Moreover, the countries that held the lowest ASDR, such as Armenia, Azerbaijan and China, had the lowest age-standardized DALYs simultaneously. Taiwan and Italy, the countries with the first and second place of EAPC of ASIR, are also the first and third place in EAPC of age-standardized DALYs (EAPC: 6.48 and 4.73 respectively) (Figure 2F, Supplementary Data Sheet 3). In Figure 3C, the SDI negatively correlated with age-standardized DALYs (R = −0.40, *P* < 0.0001), which means that the more developed region is more likely to have lower DALYs.

### Age Distribution

**Figure 4:**
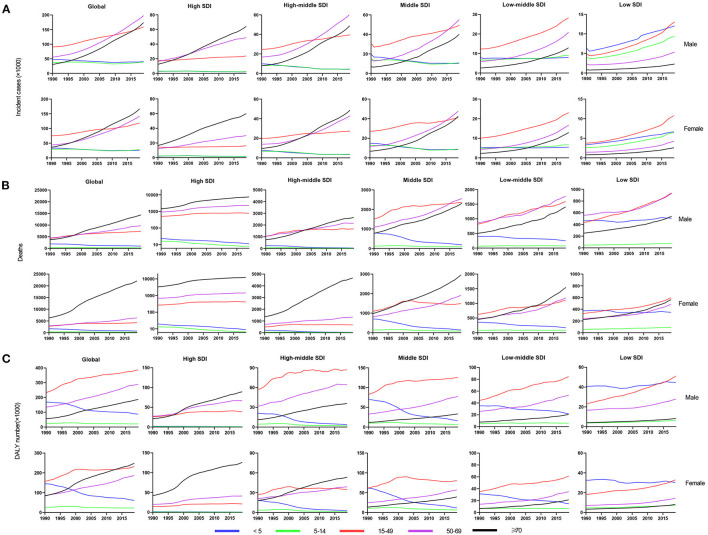
The change trends of infective endocarditis incidences, deaths and DALYs from 1990 to 2019 in different age groups. The change trends of incidences **(A)**; the change trends of deaths **(B)**; and the change trends of DALYs **(C)**. DALYs, disability-adjusted life-years; SDI, social-demographic index.

Age distribution is likewise a vital parameter of IE epidemiology. Globally, patients aged 50 years or older accounted for nearly 63% IE incidence (Figure 4A) and 79% IE mortality (Figure 4B) in 2019, significantly outnumbering the incidence and mortality in 1990 (35% and 60%, respectively). This signified the great disease burden in aged patients, especially in relatively developed regions. In contrast, patients aged under 14 years had an evident decline in IE incidence and mortality in 2019 compared with 1990 (incidence: 31 to 12%; mortality: 15–3%). However, the morbidity and mortality of those under 14 remained relatively high in low SDI regions. For DALYs, although there was an upward trend globally (Figure 4C), the age-standardized DALYs and EAPC were both decreased (Table 3). Interestingly, the IE patients aged under 5 years had a major number of DALYs in 1990 but almost decreased to the lowest in 2019, and the same changes in this age group were observed in all SDI regions. Generally, despite varying greatly in different age groups in different regions, the IE burden predominately affected the elderly and showed an alleviation in the youngers.

### The Correlation Between EAPC and SDI in 2019 or ASIR in 1990

**Figure 5:**
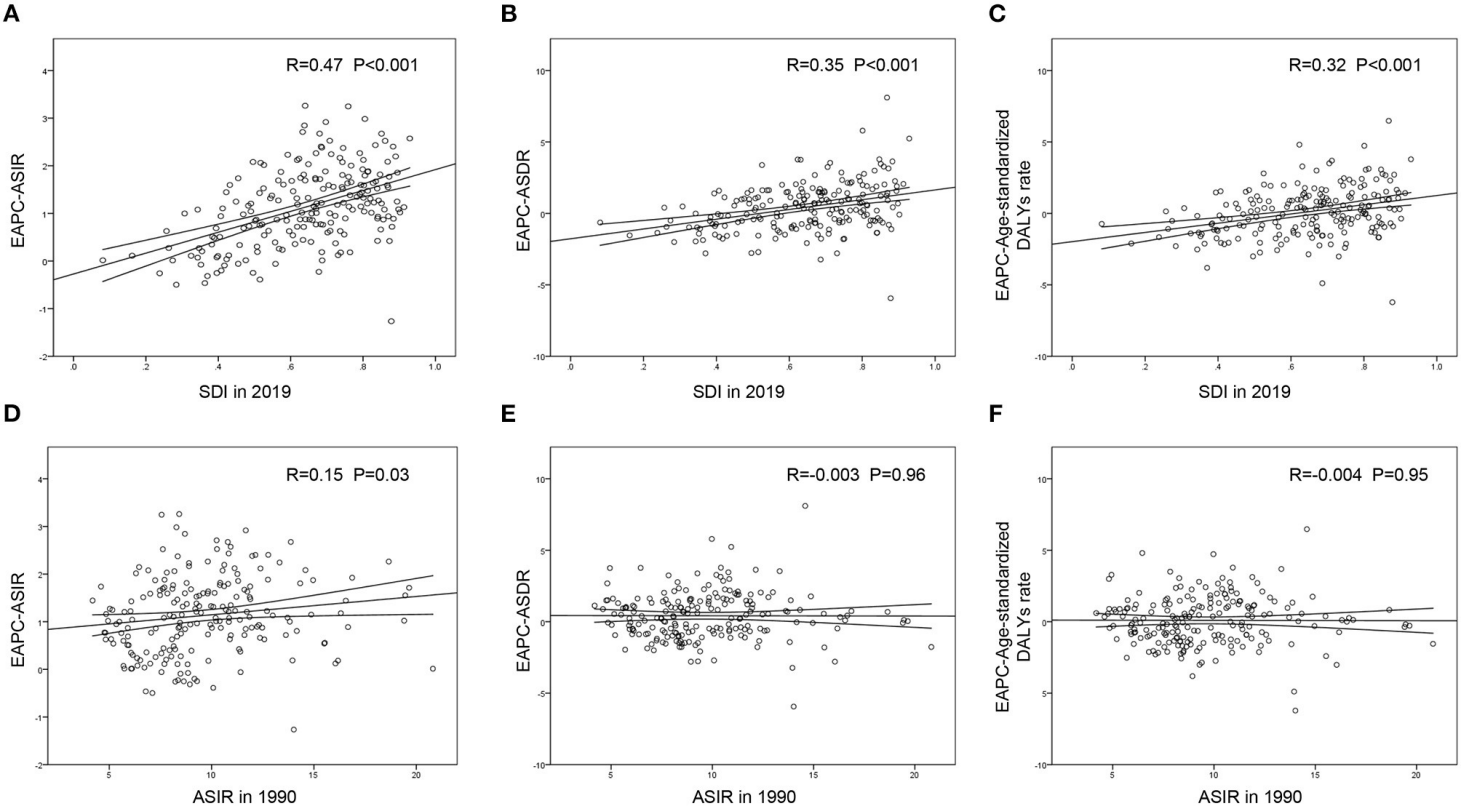
The correlation between EAPC and SDI in 2019 or ASIR in 1990 in 204 countries and territories. The SDI in 2019 was positively correlated with EPAC of ASIR **(A)**, ASDR **(B)** and age-standardized DALY rate **(C)**. The ASIR in 1990 presented little or no correlation with EPAC of ASIR **(D)**, EPAC of ASDR **(E)** or EPAC of age-standardized DALY rate **(F)**. EAPC, estimated annual percentage change; ASIR, age-standardized incident rate; ASDR, age-standardized death rate; DALYs, disability-adjusted life-years; SDI, social-demographic index.

A significant positive association was found between the SDI and EAPC in 2019, regardless of the EAPC of the ASIR (r = 0.47, *p* < 0.001), ASDR (r = 0.35, *p* < 0.001), or age-standardized DALY rate (r = 0.32, *p* < 0.001) (Figures 5A–C). These scatter plots indicate that the more developed the region is, the more likely it is to change the disease burden. Furthermore, we analyzed the association between the ASIR in 1990 and EAPC of ASRs, all presenting no distinctiveness (Figures 5D–F), which demonstrated that the baseline IE incidence in 1990 had no significant impact on annual changes in IE burden.

## Discussion

In this study, we assessed the latest infective endocarditis burden and temporal trends of incidence, mortality and DALYs in 204 countries and territories, 21 geographic areas and 5 SDI regions and analyzed the relationship between the statistics. Generally, the incidence of IE has been on the rise in most regions worldwide during the past 30 years. However, the trends of mortality and DALYs were heterogeneous across regions and countries and varied among different SDI regions. Meanwhile, the impact of gender and age on the ASIR and ASDR of IE were also prominent, with a preference for morbidity in males and elderly individuals. Furthermore, we determined that the SDI in 2019 was associated with the EAPC of these three parameters above. Therefore, the systematic understanding of global epidemiology for IE and the specific change pattern in disease burden may be valuable for public health officials and policy-makers to develop the corresponding prevention and control measures and allocate rational medical resources.

As our article showed, the cases of IE increased year by year between 1990 and 2019, as did the incidence in the vast majority of countries, corresponding to previous studies (23, 24). The noteworthy changes in the incidence and epidemiology of IE worldwide might be ascribed to multiple causes. Specifically, the modified Duke clinical diagnosis criteria incorporated more items, including clinical findings, microbiological analysis, imaging results, and available pathological specimen detections, and prosthetic valve endocarditis and cardiac device infection were also considered in IE diagnosis (8, 25), which enlarged the spectrum of disease inclusion. Moreover, the improved methods of detection in GBD study may have been in part responsible for the increase in cases and population aging aggravated the IE burden. In addition, the increase in pathogenic bacteria and mushrooming employment of cardiac indwelling electronic devices as medical technology progresses were likewise contributors to the increase in IE incidence.

Several important changes were identified in this study, including a significantly increased rate of IE in males and those of older age. In line with previous studies (26, 27), we observed that IE predominantly affected elderly individuals (aged above 50 years old), which demonstrated the potential influence of age on IE morbidity. We speculated that elderly individuals were more likely to suffer from nosocomial IE due to the greater requirements for invasive procedures. Furthermore, we found higher ASRs of incidence, death and DALYs, and faster increase in EAPC of ASIR and ASDR, as well as slower decrease in EAPC of DALYs in males during the past 30 years, which all demonstrated graver insults of IE in male health to some extent without the exact reason reported yet.

The geographical difference in IE morbidity remains obvious based on the division of socioeconomic status. The positive correlation between the ASIR and SDI suggested that the ASIR seemed to be higher in relatively developed regions. In recent decades, there have been huge changes in IE risk factors (3) which may result in epidemic differences among various SDI regions. The latest review on The Lancet (8) listed core cardiac risk factors and noncardiac risk factors, including rheumatic heart disease (RHD), prosthetic valves, intravenous drug use, cardiac indwelling electronic devices and others. Approximately 50% of these IE cases were health-care associated and constantly increased, especially for nosocomial IE, including prosthetic valve-associated IE, intravenous drug use-associated IE and cardiac indwelling electronic devices-associated IE, in America (28–30). A similar trend could be seen in other developed countries, such as Spain, Germany and Italy (31–33). Moreover, the new trend in percutaneous pulmonary valve implantation may be contributing to increased IE incidence (34). The reason why the potential pathogenic contributors to IE changed so much might be the in-mounting burden due to drugs and population aging in countries with higher SDI levels (35). Interestingly, the countries with the highest incidence of IE, namely Saint Lucia, Grenada, and Barbados, are all island nations, and the reason different from the developed countries like America etc countries is the small number of population and therefore ASIR is more likely to be affected by the number of people diagnosed with IE. For developing countries, RHD accounted for up to two-thirds of IE cases (36), even if developed countries have achieved the near elimination of acute rheumatic fever and a reduction in the rates of rheumatic heart disease during the late 20th century (37). Since RHD is a post-infection inflammation often attacking children and adolescents (38), the rate of community-acquired IE in low-SDI countries is higher, and the patients are younger. In addition, we concluded that the EAPC of the ASIR and SDI are positively correlated, which means that the influence of newly emerging disease patterns in developed countries is great and that the incidence of IE may be higher with the development of the global economy. The low EAPC of ASIR in some low SDI countries, such as Burundi and South Sudan, may be attributed to the constant decline in RHD burden globally.

Globally, the deaths due to IE were substantially mounting in 2019, especially for high SDI regions. Intractable bacteria such as Staphylococcus aureus instead of viridans group streptococci were considered the predominant pathogens for IE (26, 39). Unfortunately, Staphylococcus aureus has a notorious propensity to acquire antibiotic resistance, and meticillin-resistant strains have emerged worldwide (40), which contributes to poor prognosis and high mortality. The susceptibility to S aureus is mainly attributed to the altered causative constitution of IE, including a growing body of health-care-associated IE patients affected by Staphylococcus aureus after experiencing invasive procedures (41, 42), immunosuppressive patients with long-term intravenous drug use or suffering from carcinoma and diabetes, and population aging, especially in developed regions. But the infection to S aureus is decreased in countries that have implemented antimicrobial stewardship and limited the prescription of cephalosporins, which may decrease the IE incidence to some extent. Moreover, despite a certain increase in deaths in low-income regions, the ASDR and EAPC of ASDR presented obvious shrinkage, which was predominantly ascribed to improved medical treatment for RHD in these regions. We also found a positive association between EAPC of ASDR and SDI, meaning that the regions with higher SDI had a more distinctive change in the ASDR of IE, which was partly related to the contributing factors mentioned above.

In our paper, we showed that the global trend of IE age-standardized DALYs was declining despite variances among countries and regions. The correlation analysis revealed a negative association between the age-standardized DALY rate and SDI. Furthermore, there was an obvious positive correlation between the EAPC of age-standardized DALY rates and the SDI in 2019, which demonstrated that the higher the SDI was, the greater the change in age-standardized DALY rates was. As of now, there presents few reports explaining the changes of DALYs for IE and research is required for the exploration of the reasons behind. Here, considering that the factors possibly contributing to IE incidence and mortality were mainly nosocomial, such as prosthetic valve, intravenous drug use, and cardiac indwelling electronic devices, we ascribed the decreasing DALYs partly to the progress in health care and medical technology, which obviously promoted better IE diagnosis and therapy, improved patients' quality of life, and lessened IE DALYs. Especially in relatively developed regions and countries like America and Western European countries, there are advanced medical levels despite higher IE incidence, which is consistent with the conclusion in previous study that better quality of care for endocarditis was observed in countries with higher socio-economic status (43).

There remain some limitations in the current study. The methods of the GBD presented potential biases on our estimates in the current article, as with all GBD research. First, the limited availability and quality of surveillance data from high-burden countries was an important limitation. Moreover, the missed diagnosis and misdiagnosis in clinical practice owing to the complexity of IE increased the underreported cases. Especially in developing countries, the biased estimates were inevitable owing to the high rate of missed diagnosis and misdiagnosis. Third, differences in healthcare availability, morbidity of rheumatic heart disease, prosthetic valve, intravenous drug use, cardiac indwelling electronic devices and etc will affect the incidence of IE in different countries, and further lead to the deviated assessments for IE burden. Finally, analysis according to the clinical classification of IE requires more studies to better understand the epidemiology of IE.

In summary, the overall burden of infective endocarditis is staggering and continues to increase annually, and the incidence, mortality and disability-adjusted life years present substantial heterogeneity in different genders, ages and regions. The current study may provide a vital reference for policy-makers and clinical scientists to respond to IE and formulate more effective interventional measures.

## Data Availability Statement

The datasets presented in this study can be found in online repositories. The names of the repository/repositories and accession number(s) can be found in the article/Supplementary Material.

## Author Contributions

ZZ had full access to the data in the study and take responsibility for the integrity of the data and the accuracy of the data analysis. ZZ and HC: concept and design, revision of the manuscript, and supervision. YZ, ZC, KZ, YG, LC, and JZ: acquisition, analysis, and interpretation of data. HC, YZ, and ZC: drafting of the manuscript. HC and YZ: statistical analysis. All authors approved the final manuscript. All authors contributed to the article and approved the submitted version.

## Funding

This work was funded by National Natural Science Foundation of China (Nos. 81800041 and 82000078).

## Conflict of Interest

The authors declare that the research was conducted in the absence of any commercial or financial relationships that could be construed as a potential conflict of interest. The reviewer MY declared a shared affiliation with several of the authors HC, YZ, YG, JZ, ZC, and ZZ, to the handling editor at the time of review.

## Publisher's Note

All claims expressed in this article are solely those of the authors and do not necessarily represent those of their affiliated organizations, or those of the publisher, the editors and the reviewers. Any product that may be evaluated in this article, or claim that may be made by its manufacturer, is not guaranteed or endorsed by the publisher.

## Abbreviations

ASR: age-standardized rate
ASDR: age-standardized death rate
ASIR: age-standardized incident rate
CI: certainty interval
DALY: disability-adjusted life year
EAPC: estimated annual percentage change
GBD: global burden of disease
IE: infective endocarditis
RHD: rheumatic heart disease
SDI: social-demographic index.
