# Supplementary material for: The Global, Regional, and National Burden and Trends of Infective Endocarditis From 1990 to 2019: Results From the Global Burden of Disease Study 2019
Source: Front Med (Lausanne). 2022 Mar 9;9:774224. doi: 10.3389/fmed.2022.774224 (PMC8959916; doi:10.3389/fmed.2022.774224)
Supplement: Supplementary file 7 [file Table_1.DOCX]

GBD overview[1]
Section 1: Geographic locations of the analysis
We produced estimates for 204 countries and territories that were grouped into 21 regions and seven super-regions. The seven super-regions are central Europe, eastern Europe, and central Asia; high income; Latin America and the Caribbean; north Africa and the Middle East; south Asia; southeast Asia, east Asia, and Oceania; and sub-Saharan Africa. For GBD 2019, nine countries and territories (Cook Islands, Monaco, San Marino, Nauru, Niue, Palau, Saint Kitts and Nevis, Tokelau, and Tuvalu) were added, such that the GBD location hierarchy now includes all WHO member states. This round, GBD includes subnational analyses for several new countries and continues to analyse at subnational levels countries that were added in previous cycles. Subnational estimation in GBD 2019 includes five new countries (Italy, Nigeria, Pakistan, the Philippines, and Poland) and 16 countries previously estimated at subnational levels (Brazil, China, Ethiopia, India, Indonesia, Iran, Japan, Kenya, Mexico, New Zealand, Norway, Russia, South Africa, Sweden, the UK, and the USA). All analyses are at the first level of administrative organisation within each country except for New Zealand (by Māori ethnicity), Sweden (by Stockholm and non-Stockholm), the UK (by local government authorities), and the Philippines (by provinces). All subnational estimates for these countries were incorporated into model development and evaluation as part of GBD 2017. To meet data use requirements, in this publication we present subnational estimates for Brazil, India, Indonesia, Japan, Kenya, Mexico, Sweden, the UK, and the USA). Subnational estimates for China are included in maps but are not reported in appendix tables. Subnational estimates for other countries will be released in separate publications.

For GBD 2019, we have also defined locations as standard locations and non-standard locations. Standard GBD locations are defined as the set of all subnationals belonging to countries where data quality is high and with populations over 200 million, in addition to all other countries. Standard locations include the subnationals for China, India, the USA, and Brazil, but not Indonesia; data for China, India, the USA, and Brazil are also included at the country level. All other countries with subnational estimates are defined as non-standard locations.
Section 2: Time period of the analysis
We estimated numbers and rates of incidence, prevalence, years lived with disability (YLDs), and disability-adjusted life-years (DALYs) for the years 1990–2019; we estimated deaths and years of life lost (YLLs) for 1980–2019.
Section 3: GBD cause list
The GBD cause and sequelae list is organized hierarchically to accommodate different purposes and needs of various users. The first two levels aggregate causes into general groupings. At Level 1 there are three cause groups: communicable, maternal, neonatal, and nutritional diseases (Group 1 diseases); non-communicable diseases (Group 2); and injuries (Group 3). These Level 1 aggregates are subdivided at Level 2 of the hierarchy into 22 cause groupings (eg, neonatal disorders, neurological disorders, and transport injuries). The disaggregation into Levels 3 and 4 contains the finest level of detail for causes captured in GBD 2019. The greatest detail available for some causes, such as anxiety disorders or rheumatoid arthritis, is at Level 3 of the hierarchy, while other specific causes are at Level 4 of the hierarchy with an aggregate category at Level 3 (for example, depressive disorders at Level 3, which encompasses major depressive disorders and dysthymia at Level 4). Sequelae of diseases and injuries are organised at Levels 5 and 6 of the hierarchy. In GBD, sequelae are defined as distinct, mutually exclusive categories of healt consequences that can be directly attributed to a cause. For example, both neuropathy and blindness due to diabetic retinopathy are sequelae of diabetes; stroke and ischaemic heart disease are not, as these consequences cannot be categorically ascribed to diabetes in an individual despite good evidence for increased risk of these outcomes. The finest detail for all sequelae estimated in GBD is at Level 6 and is aggregated into summary sequelae categories (Level 5) for causes with large numbers of sequelae. Examples include the grouping of the infectious disease episodes and long-term sequelae of meningitis. For GBD 2019 there are 3473 mutually exclusive and collectively exhaustive sequela, 2063 cause sequelae and 1410 injuries sequelae, and thus our YLD estimates at each level of the hierarchy sum to the total of the level above. Prevalence and incidence aggregation are estimated at the level of individual who may have more than one sequela or disease and therefore are not additive.
The GBD causes list continues to evolve to reflect the policy relevance, and public health and medical care importance of the causes of major losses of health. The cause and sequelae list expanded based on input from the Scientific Council and GBD collaborator network. For GBD 2019, the causes of death-causes list have increased to 286 causes, from the 282 causes in GBD 2017. The non-fatal cause list has expanded from 354 causes in GBD 2017 to 364 causes in GBD 2019. The total number of fatal and non-fatal causes combined for GBD 2019 is 369. As in GBD 2017, we made no estimates for YLDs for just five causes, either because no disability is possible (as is the case with sudden infant death syndrome); because disability may occur rarely but at levels too low for accurate estimation given the data (as for aortic aneurysm); or because the disability is captured by the complicating causes that led to that cause of death (as for indirect maternal deaths, late maternal deaths, and maternal deaths aggravated by HIV/AIDS).

Section 4 GBD results overview[2; 3]
Results from GBD 2019 are available through an interactive data downloading tool on the Global Health Data Exchange (GHDx). The GHDx is the world’s most comprehensive catalogue of surveys, censuses, vital statistics, and other health-related data. Results are measured in terabytes.

The latest version of the data download tool, available here: <http://ghdx.healthdata.org/GBD-results> tool, contains core summary results for GBD 2019. These results include deaths, years of life lost (YLLs), YLDs, disability-adjusted life-years (DALYs), prevalence, incidence, and rate of change. The GHDx includes data for causes, risks, cause-risk attribution, aetiologies, and impairments.
Data above a certain size cannot be viewed online but can be downloaded. Depending on the size of the download, users may need to enter an email address; a download location will be sent to them when the files are prepared.
All GBD 2019 online data visualisations are available at <http://vizhub.healthdata.org/GBD-compare>, which provides results for all GBD health metrics.
Section 5 Data input sources overview[2]
GBD 2019 syntheses a large and growing number of data input sources including surveys, censuses, vital statistics, and other health-related data sources. The data from these sources are used to estimate morbidity; illness, and injury; and attributable risk for 204 countries and territories from 1990 to 2019; mortality deaths are estimated from 1980 to 2019. The input sources are accessible through an interactive citation tool available in the GHDx.
Citations for specific GBD components, causes and risks, and locations can be found through the Data Input Sources Tool in GHDx: http://ghdx.healthdata.org/gbd-2019/data-input-sources. This tool allows users to view and access GHDx records for input sources and export a comma-separated value (CSV) file that includes metadata, citations, and information about where the data were used in GBD. As required by GATHER, additional metadata for input sources are available through the citation tool as well.

**References**[1] G.B.D. Diseases, and C. Injuries, Global burden of 369 diseases and injuries in 204 countries and territories, 1990-2019: a systematic analysis for the Global Burden of Disease Study 2019. Lancet 396 (2020) 1204-1222.

[2] G.B.D. Mortality, and C. Causes of Death, Global, regional, and national age-sex specific all-cause and cause-specific mortality for 240 causes of death, 1990-2013: a systematic analysis for the Global Burden of Disease Study 2013. Lancet 385 (2015) 117-71.

[3] C.J. Murray, K.F. Ortblad, C. Guinovart, S.S. Lim, T.M. Wolock, D.A. Roberts, E.A. Dansereau, N. Graetz, R.M. Barber, J.C. Brown, H. Wang, H.C. Duber, M. Naghavi, D. Dicker, L. Dandona, J.A. Salomon, K.R. Heuton, K. Foreman, D.E. Phillips, T.D. Fleming, A.D. Flaxman, B.K. Phillips, E.K. Johnson, M.S. Coggeshall, F. Abd-Allah, S.F. Abera, J.P. Abraham, I. Abubakar, L.J. Abu-Raddad, N.M. Abu-Rmeileh, T. Achoki, A.O. Adeyemo, A.K. Adou, J.C. Adsuar, E.E. Agardh, D. Akena, M.J. Al Kahbouri, D. Alasfoor, M.I. Albittar, G. Alcala-Cerra, M.A. Alegretti, Z.A. Alemu, R. Alfonso-Cristancho, S. Alhabib, R. Ali, F. Alla, P.J. Allen, U. Alsharif, E. Alvarez, N. Alvis-Guzman, A.A. Amankwaa, A.T. Amare, H. Amini, W. Ammar, B.O. Anderson, C.A. Antonio, P. Anwari, J. Arnlov, V.S. Arsenijevic, A. Artaman, R.J. Asghar, R. Assadi, L.S. Atkins, A. Badawi, K. Balakrishnan, A. Banerjee, S. Basu, J. Beardsley, T. Bekele, M.L. Bell, E. Bernabe, T.J. Beyene, N. Bhala, A. Bhalla, Z.A. Bhutta, A.B. Abdulhak, A. Binagwaho, J.D. Blore, B.B. Basara, D. Bose, M. Brainin, N. Breitborde, C.A. Castaneda-Orjuela, F. Catala-Lopez, V.K. Chadha, J.C. Chang, P.P. Chiang, T.W. Chuang, M. Colomar, L.T. Cooper, C. Cooper, K.J. Courville, B.C. Cowie, M.H. Criqui, R. Dandona, A. Dayama, D. De Leo, L. Degenhardt, B. Del Pozo-Cruz, K. Deribe, et al., Global, regional, and national incidence and mortality for HIV, tuberculosis, and malaria during 1990-2013: a systematic analysis for the Global Burden of Disease Study 2013. Lancet 384 (2014) 1005-70.
